# Malignant Sinonasal Tumors: Update on Histological and Clinical Management

**DOI:** 10.3390/curroncol28040222

**Published:** 2021-07-01

**Authors:** Alessandra Bracigliano, Fabiana Tatangelo, Francesco Perri, Giuseppe Di Lorenzo, Roberto Tafuto, Alessandro Ottaiano, Ottavia Clemente, Maria Luisa Barretta, Nunzia Simona Losito, Mariachiara Santorsola, Salvatore Tafuto

**Affiliations:** 1 Nuclear Medicine Unit, Istituto Nazionale Tumori, IRCCS—Fondazione “G. Pascale”, 80131 Naples, Italy; 2 Department of Pathology, Istituto Nazionale Tumori, IRCCS—Fondazione “G. Pascale”, 80131 Naples, Italy; f.tatangelo@istitutotumori.na.it (F.T.); n.losito@istitutotumori.na.it (N.S.L.); 3 Head and Neck Oncology Unit, Istituto Nazionale Tumori, IRCCS—Fondazione “G. Pascale”, 80131 Naples, Italy; f.perri@istitutotumori.na.it; 4 Sarcomas and Rare Tumors Unit, Istituto Nazionale Tumori, IRCCS—Fondazione “G. Pascale”, 80131 Naples, Italy; giuseppedilorenzo10@gmail.com (G.D.L.); ottavia.clemente@istitutotumori.na.it (O.C.); s.tafuto@istitutotumori.na.it (S.T.); 5 Neurosurgery Unit, Federico II University, 80131 Naples, Italy; rob.tafuto@gmail.com; 6 Division of Innovative Therapies for Abdominal Metastases, Istituto Nazionale Tumori, IRCCS—Fondazione “G. Pascale”, 80131 Naples, Italy; a.ottaiano@istitutotumori.na.it (A.O.); mariachiara.santorsola@istitutotumori.na.it (M.S.); 7 Radiology Unit, Istituto Nazionale Tumori, IRCCS—Fondazione “G. Pascale”, 80131 Naples, Italy; m.barretta@istitutotumori.na.it

**Keywords:** sinonasal neuroendocrine neoplasms, tumors of sinonasal tract, ethmoid sinus salivary gland type, neuroendocrine carcinomas of the head and neck

## Abstract

Tumors of nasal cavity and paranasal sinuses (TuNSs) are rare and heterogeneous malignancies, presenting different histological features and clinical behavior. We reviewed the literature about etiology, biology, and clinical features of TuNSs to define pathologic features and possible treatment strategies. From a diagnostic point of view, it is mandatory to have high expertise and perform an immunohistochemical assessment to distinguish between different histotypes. Due to the extreme rarity of these neoplasms, there are no standard and evidence-based therapeutic strategies, lacking prospective and large clinical trials. In fact, most studies are retrospective analyses. Surgery represents the mainstay of treatment of TuNSs for small and localized tumors allowing complete tumor removal. Locally advanced lesions require more demolitive surgery that should be always followed by adjuvant radio- or chemo-radiotherapy. Recurrent/metastatic disease requires palliative chemo- and/or radiotherapy. Many studies emphasize the role of specific genes mutations in the development of TuNSs like mutations in the exons 4–9 of the TP53 gene, in the exon 9 of the PIK3CA gene and in the promoter of the TERT gene. In the near future, this genetic assessment will have new therapeutic implications. Future improvements in the understanding of the etiology, biology, and clinical features of TuNSs are warranted to improve their management.

## 1. Introduction

Sinonasal tumors (TuNSs) are a rare disease affecting fewer than 1 person out of 100,000 individuals per year worldwide. They account for less than 1% of all human malignant cancers, less than 3% of all head and neck carcinomas, and have a peak incidence in the 5th to 7th decades with a male preponderance (2:1 rate) [1,2,3,4,5,6]. TuNSs can have an epithelial (carcinomas) or mesenchymal (sarcomas) origin. In fact, substrates for their development can be the different tissues covering nasal cavities and paranasal sinuses, such as mucosal epithelium, serous gland epithelium, mesenchymal tissues, cartilage, neural/neuroectodermal tissues, hemato-lymphoid cells, and the odontogenic apparatus. Epithelial tumors are the most common form and originate from the epithelial lining, accessory salivary glands, neuroendocrine tissue, and olfactory epithelium. Conversely, mesenchymal tumors derive from the supporting tissue [7].

TuNSs are a heterogeneous category of malignancies, presenting both different histological features and clinical behavior. They should not be included in the “miscellany” of head and neck cancers, but considered as separate entities. The prognosis is dismal with a 5-year overall survival ranging from 60% for early tumors (T1–2) to 20% for advanced disease. Most newly diagnosed TuNSs are locally advanced or metastatic cancers, not suitable for radical treatments. Surgery represents the mainstay of treatment and every patient with a diagnosis of TuNSs, independently from their histology, should be evaluated by a surgeon in the context of a multidisciplinary team [8]. Unresectable tumors present a poor outcome and they are treated with palliative strategies. Understanding the etiology, biology, and clinical features of TuNSs may help the specialists to face them, since no clear treatment guidelines are available. In sinonasal imaging, it is well known that benign tumours cause slight remodeling and thickening of adjacent bone, while malignant ones destroy it. However, malignant tumours may also remodel bone rather than destroy it; e.g., sinonasal sarcomas, minor salivary gland carcinomas, extramedullary plasmacytomas, large cell lymphomas, olfactory neuroblastomas, and hemangiopericytomas. In clinical practice, the most frequent five histotypes of TuNSs include squamous cell carcinoma, lymphoepithelial carcinoma, undifferentiated nasal-sinus carcinoma (SNUC), adenocarcinomas, and neuroendocrine tumors. They present with different pathologic appearance and clinical behavior (5-year survival varies from 22 to 67%). Currently, scientific efforts are oriented to gaining better knowledge of the tumorigenic pathways and to defining alternative treatment strategies. 

## 2. Materials and Methods

We made a literature review on TuNSs using a web-based search on Pubmed Central, Scopus, Google Scholar, and Cochrane Library. The search terms used to guide the research were: “paranasal sinus and nasal cavity cancers”, “paranasal sinus cancer genetics”, “paranasal sinus cancer diagnosis”, “standard treatments for paranasal sinus cancer”, and “histological variants”.

## 3. Etiopathogenesis of TuNSs

Occupational exposure to wood dust, leather, flour, tissue, and other industrial compounds (chromium and nickel), exposure to glues, formaldehyde, and organic solvents are shown to have a causal role in the development of TuNSs in several studies. For this reason, TuNSs are officially recognized as “occupational diseases” [9,10,11,12,13,14,15]. Additional non-occupational risk factors for TuNSs development include tobacco, nasal polyposis, inverted sinusal papilloma [16,17], chronic sinusitis and, finally, radiotherapy used for the treatment of retinoblastoma, a hereditary eye tumor that generally affects children. Evidence from literature suggests that also smoking tobacco can increase the risk of sinonasal tumors [15,18,19] and, in particular, of the squamous cell subtype (SNSCC) [19,20,21]. A report [19] showed a significant increase in the risk of developing TuNSs in patients exposed to wood dust with an odds ratio (OR) of 1.72 (CI 95%: 1.16–2.56) adjusted for many relevant risk factors, including tobacco smoking. Furthermore, human papillomavirus (HPV) has been detected in about 30% of sinonasal carcinomas and high-risk HPV 16 seems to be most frequently associated with the squamous cell histotype [21]. The identification of HPV in sinonasal carcinomas has important clinical implications, since it is associated with a favorable prognosis [21,22,23,24,25]. Only a few scientific works demonstrated that benign nasal diseases may represent an additional risk for the development of nasal cavity/paranasal sinus (NCPS) or nasopharyngeal cancers, but these data need to be confirmed [26,27,28,29] (Table 1).

## 4. Pathological Classification and Histotypes

The 2012 World Health Organization (WHO) classification system divided TuNSs into different histological categories based on the site of origin and their clinical behavior [30] with 5-year overall survival ranging from 22% to 67%. TuNSs include a great variety of histologic subtypes. They can be subdivided into squamous cell carcinomas, lymphoepithelial carcinoma, neuroendocrine carcinomas or undifferentiated sinonasal carcinomas (SNUC), adenocarcinomas intestinal-type (ITAC) and not intestinal-type (NON-ITAC), adenocarcinomas salivary-type, neuroendocrine tumors (NENS), and more rarely, adenoid cystic carcinomas and olfactory neuroblastomas (esthesioneuroblastomas) [3,4,5,6] (Table 2).

### 4.1. Squamous Cell Carcinoma

Squamous cell carcinoma is the most common form of TuNSs (3%) [10,15]. It is divided into keratinizing and non-keratinizing subtypes and generally arises from the maxillary sinus and nasal cavity. Squamous cell carcinoma is associated to arsenic and welding fumes exposure and it often affects male adult subjects with a male:female ratio of 2:1 [31,32,33]. The keratinizing carcinoma does not differ from the squamocellular forms that arise at the mucous level of other head/neck sites. Recently, a possible correlation has emerged between some sinonasal squamous cell carcinomas and high-risk HPV infection (about 30% of cases) [24,25,26,27,28,34,35]. Other rare variants of squamous cell carcinoma of the nasal cavities and paranasal sinuses are papillary cancer, basaloid carcinoma, sarcomatoid carcinoma, adenosquamous carcinoma, and acantholytic cancer. Another possible variant of squamous carcinoma is the “NUT midline carcinoma” (NMC), a rare form of undifferentiated carcinoma with a clinical aggressive behavior and chromosomal rearrangements of the NUT (nuclear protein in testis) gene, at 15q14 [36,37].

### 4.2. Lymphoepithelial Carcinoma

Lymphoepithelial carcinoma is an undifferentiated carcinoma, in which the epithelial neoplastic population is accompanied by a strong lymphocyte infiltration. It is morphologically similar to the undifferentiated nasopharyngeal carcinoma; furthermore, both entities are usually positive for Epstein Barr Virus (EBV). Especially in Western countries, it is a rare histotype mainly affecting male adults. Microscopically, lymphoepithelial carcinoma is characterized by large medium-sized epithelial cells, blistering nucleus, and prominent nucleole, without evidence of keratinization. During the diagnostic phase, it is fundamental to differentiate lymphoepithelial carcinoma from multiple lymphomas and undifferentiated carcinomas through immunohistochemical analysis of cytokeratins and EBV antigens expression [38,39].

### 4.3. Undifferentiated Sinonasal Carcinoma (SNUC)

The 2005 WHO classification defined undifferentiated sinonasal carcinoma as a very aggressive, undifferentiated, and not EBV-associated carcinoma [38,39,40]. It is generally diagnosed at an advanced stage and shows an extensive invasion of adjacent anatomical structures. Histologically, it appears as undifferentiated medium-large cells neoplasia, arranged in nests and in a trabecular or solid pattern. It has a strong mitotic activity, frequent areas of necrosis, and invasion of lymph-vascular spaces. The immunohistochemical profile is characterized by the expression of cytokeratins 7, 8, and 19 [40,41,42,43]. SNUC must be distinguished from lymphoepithelial carcinoma and neuroblastoma of the olfactory tract, which is negative for the expression of cytokeratins.

### 4.4. Sinonasal Adenocarcinomas

**Figure 1:**
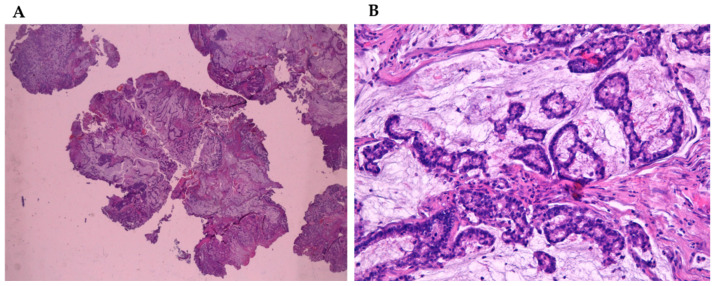
(**A**) Mucinous sinonsal adenocarcinoma (4×) stained with hematoxylin-eosin. (**B**) Mucinous sinonsal adenocarcinoma (40×) stained with hematoxylin-eosin.

Adenocarcinomas represent about 20% of the malignant neoplasms of the sinonasal tract [32] and they can arise from both the lining surface epithelium and the seromucinous glands (Figure 1A,B). WHO 2005 classification groups them into three main types, including intestinal adenocarcinoma (ITAC), non-intestinal adenocarcinomas (NO-ITAC), and salivary adenocarcinomas. Intestinal and non-intestinal types are surface-type adenocarcinomas, while salivary adenocarcinomas originate from seromucous glands of the nasal cavity and paranasal sinuses, as well as the surface epithelium. These carcinomas are similar to those originating from major and minor salivary glands [43,44,45,46]. 

### 4.5. Intestinal Adenocarcinoma

**Figure 2:**
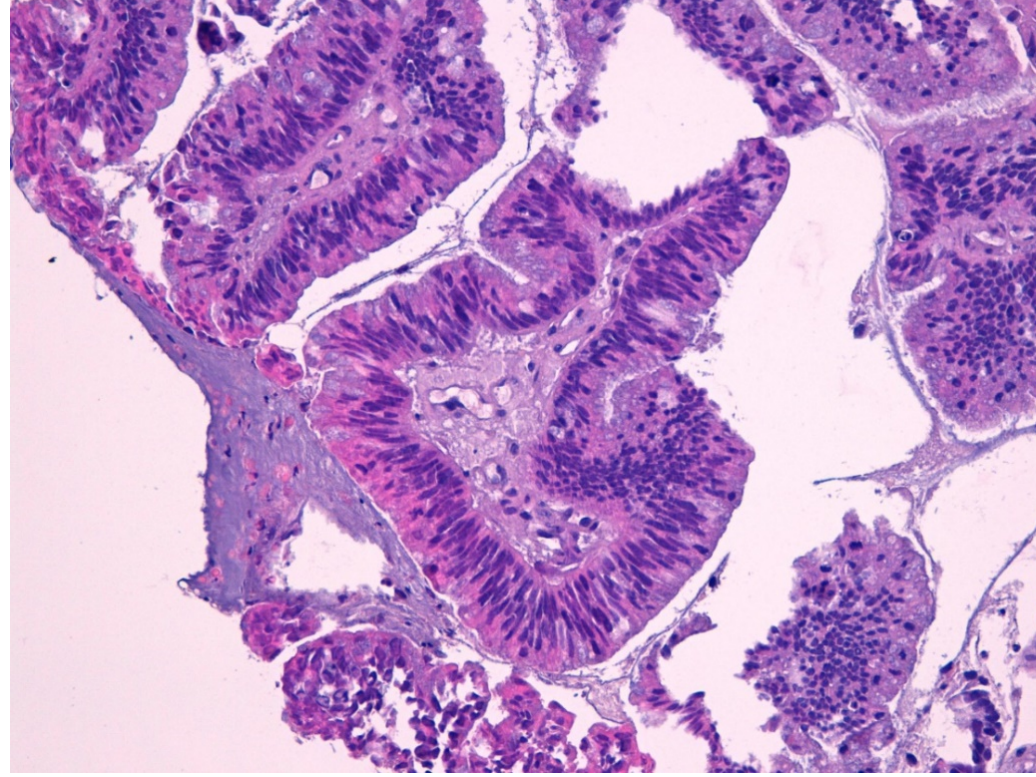
Intestinal type adenocarcinoma (40×) stained with hematoxylin-eosin.

Intestinal-type adenocarcinoma (ITAC) has histological features similar to adenoma and colorectal adenocarcinoma from which it must be differentiated (Figure 2). ITAC originates from areas of intestinal metaplasia of the Schneiderian mucosa, which covers the sinus-nasal district. It is the most frequent type of adenocarcinoma, accounting for 6% to 13% of sinonasal malignancies. It affects more frequently male subjects (male:female ratio up to 6:1) [22,43,44,45,46] with a peak in the fifth and sixth decade of life. The most frequent site is the ethmoid, followed by the nasal cavities and other paranasal sinuses.

Macroscopically, it appears more often as an exophytic, polypoid, or papillary lesion, sometimes jelly-like. Microscopically, the neoplasm is constituted by cylindrical and mucus-secreting cells. Neoplastic elements rarely show a “ring cell with bezel” appearance, due to the intracytoplasmic accumulation of mucins. Occasionally, elements with endocrine differentiation may be present. The architecture can be papillary, glandular, compact, mucinous, or mixed. The differentiation grade shows a correlation with the behavior of the neoplasm. For example, the mucinous and poorly differentiated forms, with a solid architecture, are significantly more aggressive than well-differentiated or moderately differentiated forms, with papillary or tubule-glandular architectural patterns [47]. They are also described in the literature as rare examples of ITAC combined with small cell neuroendocrine carcinoma [45]. The immune phenotype of the ITAC is characterized by positivity for cytokeratins 7 and 20 and for intestinal differentiation markers. In particular, cytokeratin 7 is frequently but not constantly positive, while cytokeratin 20 is positive in almost all cases, together with CDX2 [48,49,50,51,52]. The presence of cytological atypia, high mitotic index, and areas of necrosis are helpful in the distinction of ITAC from either benign lesions, such as mucocele, or non-intestinal type adenocarcinomas with a low grade of histological malignancy. The absence of squamous differentiation distinguishes ITAC from mucoepidermoid carcinoma and adenosquamous carcinoma [49,50,53,54]. Molecular alterations observed in ITACs are mainly mutations in the TP53 genes (60%) [54] and overexpression of EGFR or HER2 (30%) [55,56,57,58]. The rate of TP53 mutations in ITACs is about 60% and, interestingly, it is related to the duration, average and cumulative level of exposure to wood dust [53,54,55]. 

### 4.6. Non-Intestinal Adenocarcinomas

Non-intestinal sinonasal adenocarcinomas (non-ITAC) are a rare and heterogeneous group of undifferentiated tumors, sharply different from salivary histotypes. They can be further distinguished into low- and high-grade forms. More aggressive non-intestinal adenocarcinomas mainly affect nasal cavities and maxillary sinus of male subjects. Conversely, low-grade non-intestinal adenocarcinomas are located preferentially in the nasal cavities and ethmoidal sinus of adult subjects. Histological studies show that they are positive for cytokeratin 7, but not for cytokeratin 20 and CDX2 [59,60,61]. Furthermore, papillary glandular, mucinous, trabecular, cribriform, clear cells (renal carcinoma type) pattern of growth can be identified [61,62,63,64]. In any case, these are well-differentiated cancers, with absent or mild atypia, and low mitotic index. In fact, they need to be distinguished from benign lesions, such as adenomatoid epithelial respiratory hamartoma and mucinous serum hamartoma.

### 4.7. Salivary Adenocarcinomas

**Figure 3:**
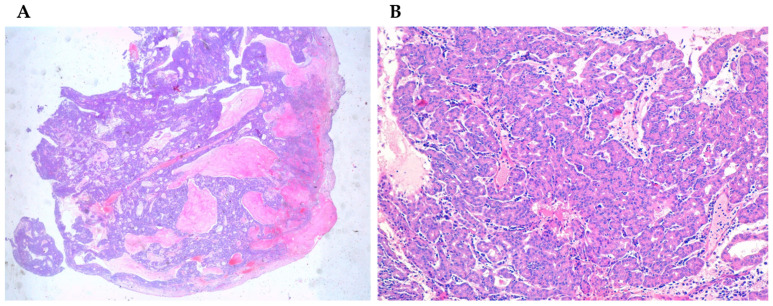
(**A**) Acinic cell carcinoma (4×) stained with hematoxylin-eosin. (**B**) Acinic cell carcinoma (40×) stained with hematoxylin-eosin.

**Figure 4:**
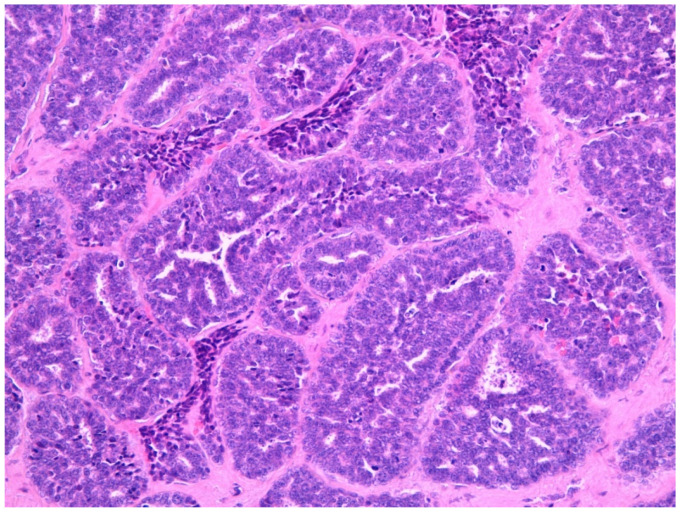
Adenoid cystic carcinona (40×) stained with hematoxylin-eosin.

Salivary adenocarcinomas originate from seromucous glands of the nasal cavity and paranasal sinuses, as well as from the surface epithelium. These histotypes are identical to the forms arising at the level of the minor and major salivary glands of the oral cavity. They show a cribriform architecture in about 50% of the cases, while in the remaining cases they have a solid or tubular architecture. Bone and nerve sheath invasion can be frequently observed. Sinonasal salivary-type carcinomas include different tumor types such as adenoid cystic carcinoma, mucoepidermoid carcinoma, acinic cell carcinoma (Figure 3A,B), myoepithelial carcinoma, epithelial-myoepithelial carcinoma, polymorphous low-grade adenocarcinoma, and carcinoma ex-pleomorphic adenoma [62,65,66]. Adenoid cystic carcinoma (Figure 4) is the most frequent histology, accounting for about 10% to 18% of TuNSs, and it is preferentially located at the level of the maxillary sinus and nasal cavities [62,65,66,67,68]. HPV infections sustained by HPV33 and HPV35 are frequently associated with its onset [35,62,69,70]. However, given the small number of cases studied so far, these findings need to be confirmed further. The less common form is the mucoepidermoid carcinoma, representing about 5% of sinonasal glandular tumors [62,71].

### 4.8. Neuroendocrine Tumors (NENs)

Primary TuNSs with neuroendocrine differentiation (SCND or Sinonasal Neuroendocrine Tumors-NENs) are infrequent tumors with histologic features similar to neuroendocrine carcinomas arising in other sites [72,73,74]. The classification is based on mitotic activity, necrosis, and nuclear pleomorphism. Sinonasal neuroendocrine tumors are divided into well- and moderately differentiated, (grade 1 and grade 2, SNECs) and undifferentiated (grade 3 neuroendocrine carcinoma, SNUC). Moreover, SNECs are sub-divided into small cell (SCNECs) and large cell (LCNECs) carcinomas. The use of the Ki-67 labeling index has been suggested as an additional objective criterion [13,75,76]. In general, conventional microscopy is not sufficient to make a definitive diagnosis, thus, immunohistochemistry studies are required. Sinonasal neuroendocrine carcinomas are usually strongly positive for synaptophysin, specific neuronal enolase (NSE), and CD56, showing a weak reaction for chromogranin and CAM5.2. The histomorphological diagnosis, along with grading and staging assessments, is crucial in predicting the prognosis of these tumors. The prognosis of sinonasal neuroendocrine carcinoma closely depends on the tumor type and grade, with high-grade carcinomas displaying the worse outcome. Sinonasal neuroendocrine carcinomas must be distinguished from olfactory neuroblastoma (ONB). The later is a neuroectodermal neoplasia presenting very similar histological features with neuroendocrine forms, especially in poorly differentiated histotypes. However, the sustentacular cells of an olfactory neuroblastoma are negative for cytokeratins (at different molecular weights) and positive for the S100 cellular protein.

### 4.9. Olfactory Neuroblastoma (ONB)

Olfactory neuroblastoma (ONB), also known as esthesioneuroblastoma, is a rare malignant tumor arising from the olfactory neuroepithelium of the superior nasal cavity. First described by Berger in 1924, it accounts for approximately 2–3% of tumors of the nasal cavity, without any race or gender predilection. It is divided by the Hyams’ histologic grading system in low-grade (Hyams I-II) and high-grade (Hyams III-IV) tumors, the first characterized by a lobular architecture with a minimal or absent mitotic activity and the presence of pseudorosettes, the second by the gradual loss of the lobular architecture, a more represented mitotic activity, and the emergence of necrosis. Molecular analysis shows overexpression of the Drosophila achaete-scute gene (hASH1) involved in immature olfactory neuronal development and in neuroendocrine differentiation. Prognostic factors and specific guidelines for ONB treatment are not well-defined, mainly due to the rarity of these neoplasms and the scarcity of studies with large case numbers. So far, treatment schemes include surgery, radiation therapy, and chemotherapy (as adjuvant or neoadjuvant) in various combinations [77].

## 5. Genetic Background of Paranasal Sinus Carcinomas

The carcinogenesis of TuNSs, regardless of their histology, depends on exposure to many different risk factors (see above Table 1). They do not have a direct carcinogenesis mechanism, as they are not mutagenic. The main carcinogenetic mechanism is the stimulation of a chronic and sustained inflammatory status. A complete dissertation of links between cancer and chronic inflammation is beyond the scope of this review. However, the continuous production of IFNα and IL-1β by lymphocytes and/or inflammatory infiltrate cells stimulates the transcription factor nuclear factor κB (NFκB) pathway. The latter pathway is associated with cell proliferation and cell cycle dysregulation within epithelial cells [78]. Another mechanism is the uncontrolled production of reactive oxygen species (ROS) which are overproduced in chronic inflammation. They cause direct damage of DNA (inactivation of tumor suppressor genes and/or the activation of oncogenes), facilitates neoplastic progression [79].

TuNSs present specific genetic signatures and altered signaling pathways. The most frequent mutated gene is TP53 (60% of TuNSs). The frequency of TP53 mutations is even more high in specific histotypes from ITACs (80%). Most of the mutations are missense with loss or attenuation of function and overexpression at IHC of the related protein. Interestingly, chronic exposure to wood and leather dust, through sustained inflammation, causes mutations that preferentially affect TP53 [80,81]. Another change frequently found in TuNSs is related to the Wnt/β-catenin pathway. Under normal conditions, β-catenin interacts with receptors of the APC family (adenomatous polyposis of the colon) and this interaction leads to the ubiquitination of the β-catenin. In the presence of overexpressed Wnt, β-catenin mainly interacts with it and switches on translocating into the nucleus. In nucleus, it acts as a transcription factor, activating the genes encoding for Cycline D1 and C- Myc, finally causing cell cycle dysregulation. Activating mutations of Wnt are detected in 30–50% of the paranasal sinus tumors [82,83]. Furthermore, about 20–30% of TuNSs are characterized by the overexpression of EGFR (epithelial growth factor receptor) at the protein level. This overexpression correlates either with amplification of the EGFR gene or with hyper-activating point mutations [84]. Often EGFR overexpression is mutually exclusive with overexpression of the p16 protein, which in turn correlates with HPV infection. Two prognostic groups can be identified: p16-positive/EGFR-negative TuNSs associated with better prognosis and p16-negative/EGFR-positive TuNSs associated with worse prognosis. These molecular features are also common for several tumors of the head and neck area [85]. Finally, on a genetic basis, SNUCs can be divided into: NUT (midline) carcinoma, SMARB1-deficient carcinoma, SMARCA4-deficient carcinoma and IDH-mutant carcinoma. The NUT carcinoma is characterized by the NUT-BRD4 gene fusion, with the generation of a chimeric protein able to dysregulate the cell cycle. SMARB1 is a tumor suppressor gene which once mutated is inactivated with consequent loss of function leading to a cell cycle dysregulation. SMARCA4 is another tumor suppressor gene whose function is very similar to that of SMARB1, namely, it is involved in the chromatin assembly. Loss of function of SMARCA4 characterizes the SMARCA4-carcinomas. IDH (isocitrate dehydrogenase) is an enzyme involved in metabolism. In normal conditions, this enzyme degrades isocitrate into hydroxyglutarate, a nutrient used to generate energy for the cell. When mutated, the aberrant enzymatic function of isocitrate dehydrogenase leads to an overproduction of hydroxyglutarate, a potent oncometabolite. This oncometabolite is translocated to the nucleus, where it causes widespread hypermethylation in gene promoter regions, thus silencing genes involved in cell differentiation and regulation of proliferation [86,87].

## 6. Clinical Aspects and Prognosis

TuNSs generally do not show specific symptoms to allow for an early diagnosis. In fact, they are often discovered during medical examinations carried out for other reasons. These tumors usually grow locally, extending to nearby structures (e.g., orbit, oral cavity, nasopharynx, and cranial base) and rarely to neck lymph nodes (especially submandibular, latero-cervical lymph nodes). They have scarce ability to spread at distant organs, however, less differentiated types (such as poorly differentiated and undifferentiated carcinoma) are more prone to metastasis. Sometimes, they become symptomatic when they extend to surrounding tissues with worsening abnormal nasal congestion, obstruction of a nostril, persistent epistaxis, nasal muchorrea, anosmia, abnormal protrusion of the eyeball, diplopia or loss of vision, ear pain or compression feeling, continuous tearing, headache, masses into nose and/or palate, neck lymph node enlargement, unexplained face distortions, etc. (Table 3). Prognostic factors include age, performance status, tumor location and local extension, histotype, presence or lack of perineural invasion. For example, about the precise site of origin, carcinomas arising from the nasal cavity show a better prognosis than those from the paranasal sinuses, likely because nasal carcinomas give rise to symptoms (e.g., nasal obstruction or epistaxis) coming earlier to clinical attention [1,2,3,4,5,10,14,16,17]. In addition, among maxillary sinus carcinomas, those arising from the anterior-inferior portion have a better prognosis than those arising from the superior-posterior portion, likely because the latter group has easier access to structures such as the orbit or skull base. Regarding staging, in patients with T1 disease, the 5-year survival rate is 80%, while in patients with T4 tumors, survival decreases to 30%. Extensive local disease involving nasopharynx, skull base, or cavernous sinuses markedly increases both surgical morbidity and local recurrence within 2 years [78,79,80,81]. In particular, maxillary sinus tumors are associated with 30–70% survival rate at 5 years after appropriate surgical excision; however, this value quickly drops to 10–20% in the case of unresectable disease [55,88,89,90,91]. For ITAC, the probability to be alive at 5 years is about 50% after surgery and postoperative chemo-radiotherapy. These tumors usually grow locally, destroying nearby structures. They rarely involve locoregional lymph nodes (such as submandibular and laterocervical nodes) and even less frequently give distant metastases which are more typical of the undifferentiated tumor.

## 7. Treatment Option Overview

**Figure 5:**
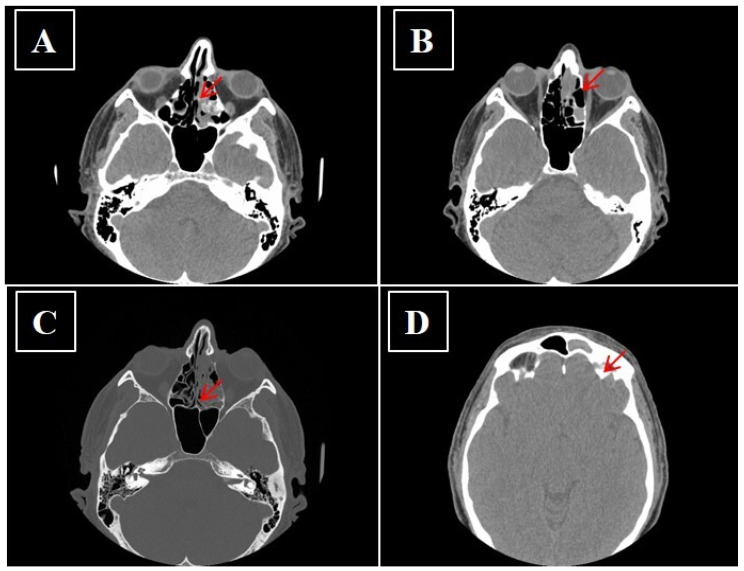
(**A**–**D**) No-contrast media CT showing sinonasal neuroendocrine tumors—MENs of ethmoidal cells and to left frontal sinus before surgery (arrow).

**Figure 6:**
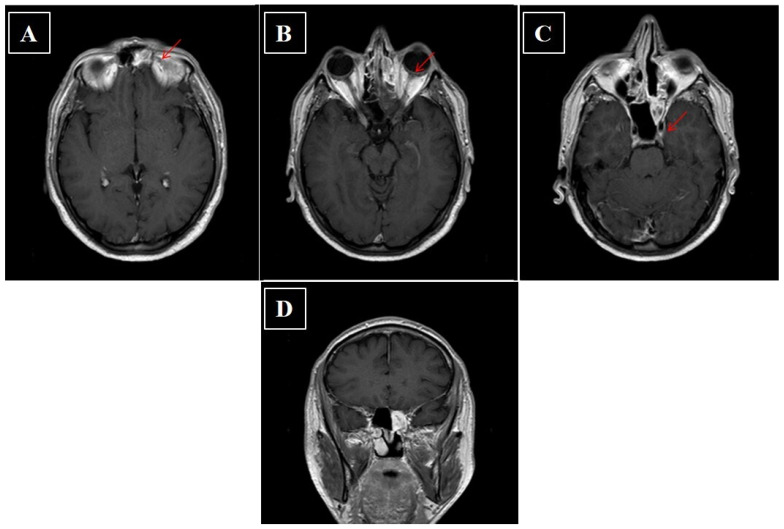
(**A**–**D**) Axial and coronal contrast enhanced fat-suppressed T1-weighted image CT scan of head performed after surgery. The arrow indicates persistence of inflammatory tissue.

**Figure 7:**
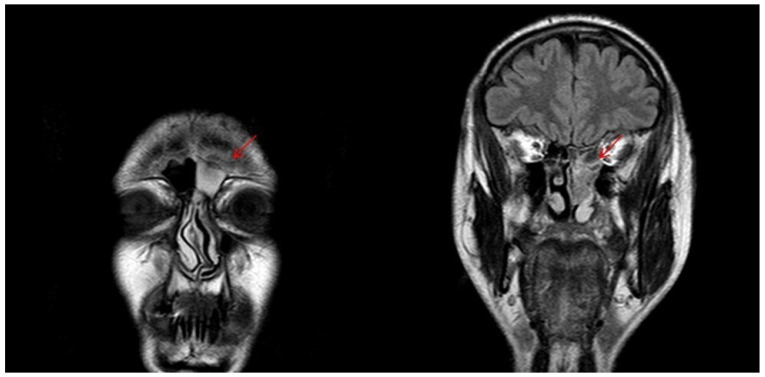
T2-weighted MRI image showing sinonasal neuroendocrine tumors—MENs of ethmoidal cells and to left frontal sinus before radiotherapy.

**Figure 8:**
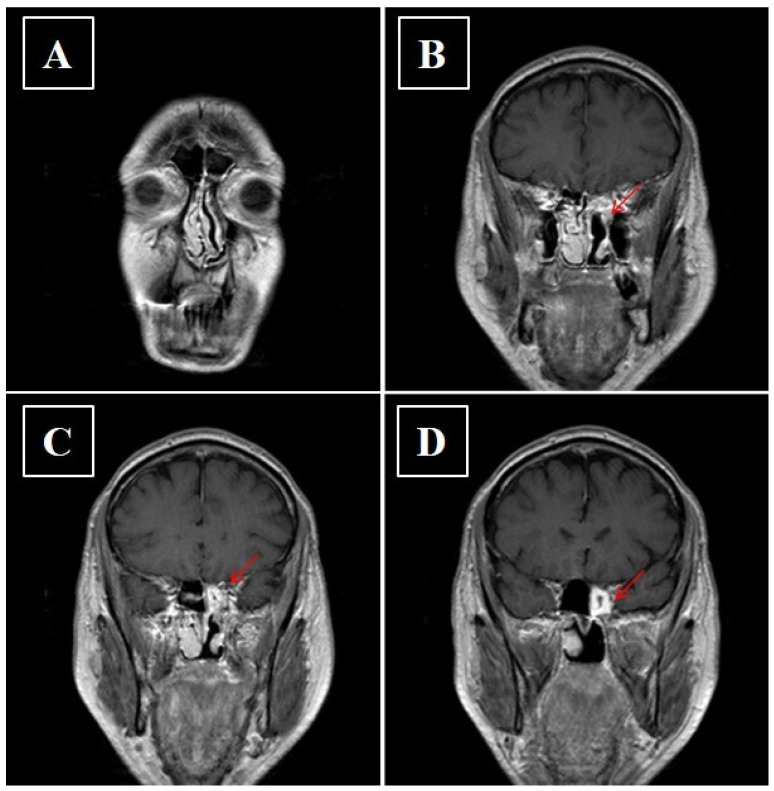
(**A**–**D**) Axial contrast enhanced fat-suppressed T1-weighted image showing reduction of the pathological tissue component in the upper offshoot of the ethmoid after adjuvant radiotherapy.

Surgery represents the mainstay of the treatment. However, the diagnosis is generally made in late phases when the tumor has infiltrated local structures and surgical removal is challenging. Treatment should be individualized based on location and extent of disease, patient performance status, histopathologic subtype, and availability of local expertise. Due to the rarity of these tumors, patients should be referred to centers with high expertise in their management. Studies have shown that surgery gives fairly good results. Surgery produces excellent control rates for T1 and T2 tumors (Figure 5A–D and Figure 6A–D). In this context, every lesion should be evaluated by the surgeon. In fact, for small and localized tumors, surgery can achieve R0 margins allowing the entire tumor’s removal, even by less invasive methods (for example, by endoscopic access). However, in most cases (including T2 tumors or adenoid cystic carcinoma), RT is given postoperatively (Figure 7 and Figure 8A–D) even if the resection margins are negative (R0). The volume to be irradiated should include the “tumor bed”, but also the neighboring lymph node levels. In fact, in some situations, such as infiltration of the pre-vertebral fascia, infiltration of the parotid and poorly differentiated tumors, prophylactic irradiation of the lymph nodes, even if clinically negative, should be considered. Adjuvant chemo-radiotherapy should be considered in presence of an involved or inadequate resection margin (R1). Notably, radical surgery, particularly in bulky tumors, should be always followed by reconstruction techniques. Radical neck dissection (levels I–V according to Robbins classification) or elective radiation therapy of the whole neck is prescribed only for patients presenting with positive neck nodes (N+). The incidence of lymph node metastases is generally low (approximately 20% of all cases). Maxillary sinus and ethmoid sinus tumors in most cases present as locally advanced disease (large T3 or T4) and they are commonly managed with surgery and postoperative chemo-radiation therapy. Cancers of the skull base, nasopharynx, or sphenoid sinus very often cannot be surgically removed. Concurrent chemo-radiation therapy may also be used for patients with comorbidities contraindicating surgery. Cisplatin or carboplatin single-agent with external beam radiation can be used in locally advanced and unresectable squamous cell carcinomas. In this case, last-generation radiotherapy techniques may be applied, such as Intensity Modulated Radiation Therapy (IMRT) and hadrontherapy [90,91,92,93,94,95,96]. Some histologies, such as ITAC and adenoid-cystic, are scarcely responsive to chemotherapy; in these cases, the use of radiotherapy should be considered. At this regard, data concerning the use of proton therapy in TuNSs (from one-institutional retrospective studies and systematic reviews) indicate both a better efficacy and activity of this therapy, compared to photon radiotherapy [97,98]. In summary, radio- and chemotherapy should be taken into account postoperatively as adjuvant treatments based on clinical and pathological risk factors. By contrast, locally advanced lesions often require more demolitive surgery and the radical resection should be always followed by adjuvant radio- or chemo-radiotherapy. If surgery is not feasible due to the extent of the disease, or to the presence of severe comorbidities, radiotherapy remains a curative treatment option, and whenever possible, it should be accompanied with chemotherapy. Notably, data from the literature show significant improvement of survival in patients treated with the combination of two or more multidisciplinary approaches (surgery, chemotherapy, and radiotherapy). Locally advanced/unresectable disease can still be approached with a curative intent attitude. In fact, even if not the best choice, sequential chemo-radiotherapy treatment, or alternatively, induction chemotherapy followed by surgery, should be evaluated. The chemotherapy regimens used in clinical practice are the same as those used for squamous neoplasms of the head and neck area and include combinations of platinum, fluoropyrimidines, and taxanes [99,100]. For recurrent/metastatic disease, the objective is to obtain disease long-term control throughout palliative chemo- and/or radiotherapy. The latter should be used for treating symptomatic sites or those at risk of bleeding or fracture. 

## 8. Conclusions

TuNSs are rare cancers, with a prevalence of much less than 1% in the general population. Well-validated therapeutic strategies are lacking as it is difficult to carry out randomized clinical trials. In general, a multimodal and multidisciplinary approach should always be pursued to overcome this gap. In the near future, both diagnosis and treatment of TuNSs will be improved through an increasing amelioration of their genetic assessment. In particular, the identification of druggable “key driver” mutations will revolutionize the treatment and change the natural history of the disease.

## Figures and Tables

**Table 1:** Sinonasal carcinoma risk factors.

Major Risk Factors	Minor Risk Factors
- inhalation of dust produced during the processing of wood, leather, flour, textiles, or nickel and chromium dust; - cigarette smoke.	- Human papillomavirus infections; - Radiotherapy carried out for the treatment of retinoblastoma; - Glue, formaldehyde, organic solvents, etc.

**Table 2:** Sinonasal neuroendocrine tumor classification.

Malignant Epithelial Tumors	Benign Epithelial Tumors
Squamous cell carcinoma	Sinonasal papillomas
○ Verrucous carcinoma	○ Inverted papilloma
○ Papillary squamous cell carcinoma	○ (Schneiderian papilloma, inverted type)
○ Basaloid squamous cell carcinoma	○ Oncocytic papilloma
○ Spindle cell carcinoma	○ (Schneiderian papilloma, oncocytic type)
○ Adenosquamous carcinoma	○ Exophytic papilloma
○ Acantholytic squamous cell carcinoma	○ (Schneiderian papilloma, exophytic type)
Lymphoepithelial carcinoma	Salivary gland-type adenomas
Sinonasal undifferentiated carcinoma	○ Pleomorphic adenoma
Adenocarcinoma	○ Myoepithelioma
○ Intestinal-type adenocarcinoma	○ Oncocytoma
○ Nonintestinal-type adenocarcinoma	
Salivary gland-type carcinomas	
○ Adenoid cystic carcinoma	
○ Acinic cell carcinoma	
○ Mucoepidermoid carcinoma	
○ Epithelial-myoepithelial carcinoma	
○ Clear cell carcinoma N.O.S.	
○ Myoepithelial carcinoma	
○ Carcinoma ex pleomorphic adenoma	
○ Polymorphous low-grade adenocarcinoma	
Neuroendocrine tumors	
○ Typical carcinoid	
○ Atypical carcinoid	
○ Small cell carcinoma, neuroendocrine type	
Soft tissue tumors	Tumors of bone and cartilage
Malignant tumors	Malignant tumors
○ Fibrosarcoma	○ Chondrosarcoma
○ Malignant fibrous histiocytoma	○ Mesenchymal chondrosarcoma
○ Leiomyosarcoma	○ Osteosarcoma
○ Rhabdomyosarcoma	○ Chordoma
○ Angiosarcoma	Benign tumors
○ Malignant peripheral nerve sheath tumor	○ Giant cell lesion
Borderline and low malignant potential tumors	○ Giant cell tumor
○ Desmoid-type fibromatosis	○ Chondroma
○ Inflammatory myofibroblastic tumor	○ Osteoma
○ Glomangiopericytoma	○ Chondroblastoma
○ (Sinonasal-type haemangiopericytoma)	○ Chondromyxoid fibroma
○ Extrapleural solitary fibrous tumor	○ Osteochondroma (exostosis)
Benign tumors	○ Osteoid osteoma
○ Myxoma	○ Osteoblastoma
○ Leiomyoma	○ Ameloblastoma
○ Haemangioma	○ Nasal chondromesenchymal hamartoma
○ Schwannoma	
○ Neurofibroma	
○ Meningioma	
Haematolymphoid tumors	Neuroectodermal
Extranodal NK/T cell lymphoma	Ewing sarcoma
Diffuse large B-cell lymphoma	Primitive neuroectodermal tumor
Extramedullary plasmacytoma	Olfactory neuroblastoma
Extramedullary myeloid sarcoma	Melanotic neuroectodermal tumor of infancy
Histiocytic sarcoma	Mucosal malignant melanoma
Langerhans cell histiocytosis	
Germ cell tumors	Secondary tumors
Immature teratoma	
Teratoma with malignant transformation	
Sinonasal yolk sac tumor (endodermal sinus tumor)	
Sinonasal teratocarcinosarcoma	
Mature teratoma	
Dermoid cyst	

**Table 3:** Sinonasal neuroendocrine tumors: common symptoms.

Symptoms
- Nasal congestion that does not improve;
- Obstruction of a nostril;
- Persistent loss of blood, mucus, or pus from the nose;
- Pain in the region around the eye;
- Anomalous protrusion of an eyeball;
- Diminished sense of smell;
- Continuous tearing;
Change in vision, a headache never experienced before, mass formation in the nose/palate.
